# Pathology-Dependent Effects Linked to Small Heat Shock Proteins Expression: An Update

**DOI:** 10.6064/2012/185641

**Published:** 2012-10-09

**Authors:** A.-P. Arrigo

**Affiliations:** Apoptosis Cancer and Development Laboratory, Lyon Cancer Research Center, INSERM U1052-CNRS UMR5286, Centre Léon Bérard, Claude Bernard University Lyon1, 28 Rue Laennec, 69008 Lyon, France

## Abstract

Small heat shock proteins (small Hsps) are stress-induced molecular chaperones that act as holdases towards polypeptides that have lost their folding in stress conditions or consequently of mutations in their coding sequence. A cellular protection against the deleterious effects mediated by damaged proteins is thus provided to cells. These chaperones are also highly expressed in response to protein conformational and inflammatory diseases and cancer pathologies. Through specific and reversible modifications in their phospho-oligomeric organization, small Hsps can chaperone appropriate client proteins in order to provide cells with resistance to different types of injuries or pathological conditions. By helping cells to better cope with their pathological status, their expression can be either beneficial, such as in diseases characterized by pathological cell degeneration, or deleterious when they are required for tumor cell survival. Moreover, small Hsps are actively released by cells and can act as immunogenic molecules that have dual effects depending on the pathology. The cellular consequences linked to their expression levels and relationships with other Hsps as well as therapeutic strategies are discussed in view of their dynamic structural organization required to interact with specific client polypeptides.

## 1. Introduction

In the early sixties, Ritossa published papers reporting that the pattern of puffing in Drosophila chromosomes was drastically altered when third instar larvae were exposed to sublethal temperatures (35°C) or to the metabolic uncoupler dinitrophenol [1, 2]. This discovery, in addition of being the first illustration that environmental changes could modify the structure of chromosomes, suggested that new RNA messengers encoding polypeptides were synthesized in response to insults. Ten years later, these proteins were identified by Tissiéres et al. [3] and called heat shock proteins (Hsps). Thereafter, this cellular response was shown to be conserved from bacteria to human, including plants, and to be triggered by many environmental stress conditions such as starvation, exercise, recovery from hypoxia, infection, UV light, inflammation and nitrogen deficiency as well as toxins (arsenic, alcohols, metals, metabolic uncouplers, anticancer drugs, and many others). This led to the conclusion that a strong positive correlation exists between the presence of heat shock proteins and the ability of organisms to withstand stress and to transiently develop resistance [4–7]. In view of these observations, Hsps were also referred to as stress proteins, and their expression is now part of the so-called cellular stress response [7]. Five families of Hsps are induced by stress: the 70 kDa (HspA-Hsp70) family, the 20–30 kDa (HspB-small Hsps, sHsps) family, the 90 kDa (HspC-Hsp90) family, the 60 kDa (HspD-Hsp60) family, and the HspH (large Hsps) family [8]. Studies were then oriented to respond to two major questions: what is the mechanism of induction of Hsps and what is their role in the stressed cell? Stress-induced transcription of Hsps genes was rapidly found to depend on the activation of a particular transcription factor called heat shock factor 1 (HSF1). Indeed, following posttranslational modifications and homotrimer formation [9, 10], cytoplasmic HSF1 is activated [11] and migrates into the nucleus to induce a massive transcription of Hsp genes [12, 13]. Towards the second question, investigators discovered that the common denominator to the different conditions and agents that induce the expression of Hsps was their ability to alter the folding of proteins, particularly newly synthesized polypeptides that are in the process of being folded [6, 14, 15]. On a more general point of view, Hsps are expressed when the cellular environment becomes deleterious and disturbs the tertiary structure of polypeptides. So, numerous conditions and agents can induce Hsps synthesis. It was then shown that Hsps are molecular chaperones [16–18] that attenuate protein folding alterations during stress and allow amplified levels of repair and refolding of damaged polypeptides during stress recovery [6, 7]. Hence, Hsps protect proteins and help them to regain a functional tertiary structure without inducing any structural alterations. The next finding was the intriguing observation that Hsps are also constitutively expressed, that is, in the absence of apparent stress conditions (as, e.g., during cell growth, differentiation, and aging), and can act as specialized chaperones in different molecular mechanisms, such as those regulating intracellular transport, cytoskeleton architecture, intracellular redox status, stabilization of specific polypeptides, and protection against spontaneous or stimulated cell death [19]. Moreover, as described below, high levels of Hsps expression is common to many pathological conditions. Taken together, these facts open a road for new medical investigations leading to a recent explosive growth of the published studies dealing with heat shock proteins in human diseases.

**Figure 1:**
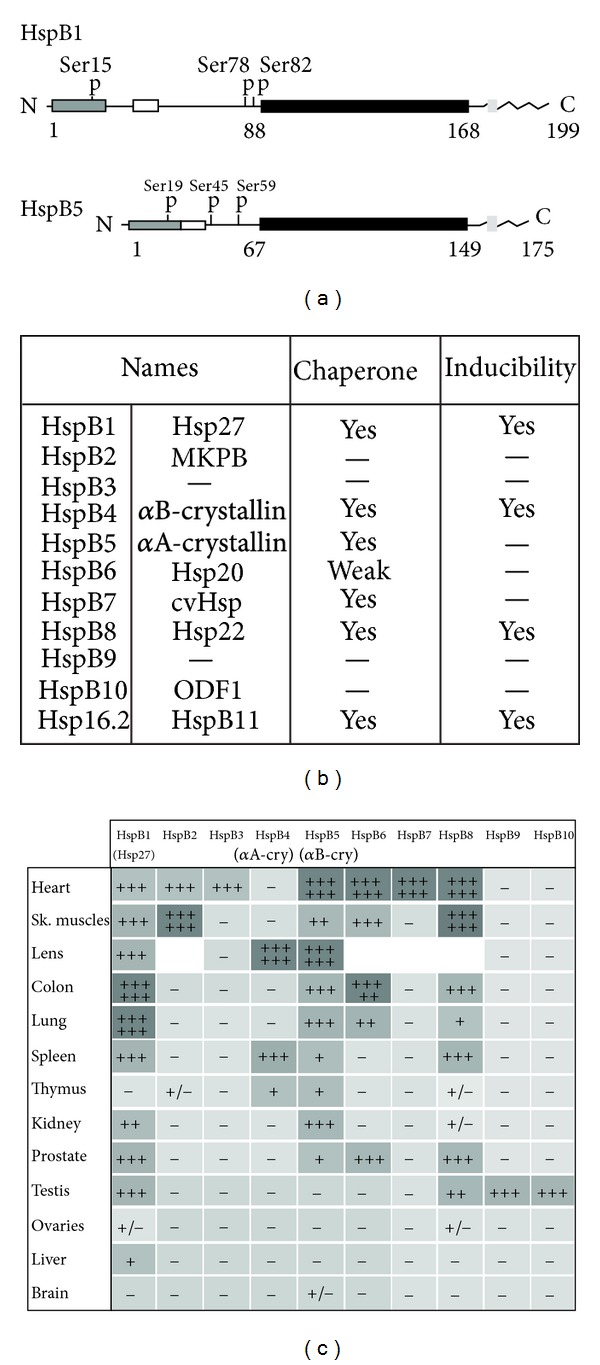
Small Hsps. (a) Organization of human HspB1 (Hsp27) and HspB5 (*α*B-crystallin) protein sequences. N-terminal gray box: WD/EPF domain; N-terminal white box: conserved sequence; black box: alpha crystallin domain; C-terminal gray box: IXI/V motif; the C-terminal flexible domain is also indicated. P: phosphorylated serine residues. Amino acids number is indicated. (b) Description of the members of the human small heat shock family of proteins. The distant Hsp16.2 polypeptide contains only a fraction of the alpha-crystallin domain. Chaperone activity and inducibility by heat shock are indicated. (c) Tissue-specific expression of constitutive small Hsps (compilation of murine as well as some human data).

Amongst Hsps, a subfamily of polypeptides in the 20–30 kDa range is characterized by the group of small stress proteins or small Hsps (HspB polypeptides) (Figure 1(a)). These proteins share a C-terminal domain in their sequence (about 40% of the proteins) which is also found in the major protein of mammalian crystallin: the alphaB-crystallin polypeptide [19–21], a less conserved N-terminal domain decorated with an hydrophobic WD/PF motif and phospho-serine sites [22], and a flexible C-terminal tail [23] containing a IXI/V motif [24]. Small Hsps also share the property to form large oligomeric structures (200–800 kDa) [19]. The human family of small Hsps contains ten members (HspB1 to HspB10) [25] plus the less conserved Hsp16.2 polypeptide [26] (see Figure 1(b)). Only four of them (HspB1, HspB5, HspB8, and HspB11) are induced by heat shock or other types of stress and five (HspB1, HspB4, HspB5, HspB8, and HspB11) bear a conserved ATP-independent chaperone activity [27, 28]. In this regard, up to now, the most studied chaperones have been HspB1 (also denoted Hsp27 or Hsp28) and the alphaA- and alphaB-crystallin polypeptides (HspB4 and HspB5). This paper discusses the multiple roles of these small Hsps in human diseases. 

## 2. Small Hsps Are Protective Molecular Chaperones towards Environmental Conditions or Agents That Alter Protein Conformation Homeostasis 

As of today, the molecular function of several high molecular weight Hsps (Hsp70, Hsp90, Hsp60) is well documented (i.e., ATP-dependent chaperones), while that of the small Hsps was, until recently, more confuse in spite of the property of some of them to act as ATP-independent chaperones [29, 30]. In stress conditions, such as heat shock, small Hsps accumulate in order to trap and store stress-altered polypeptides in a refolding competent state that can interfere with their propensity to aggregate [26, 29, 31–34]. The name“holdase” has been proposed for this intriguing activity which depends on the dynamic oligomerization/phosphorylation status of small Hsps [30, 35–39]. Indeed, subsequently to stress-induced disruption of their oligomeric distribution, these Hsps interact with stress-altered polypeptides and store them *via* the reformation of large oligomeric structures [40–43]. By doing so, the large oligomeric complexes (up to 800 kDa, in case of HspB1) act as reservoirs that can further increase their sizes if more nonnative proteins accumulate. Stored polypeptides are in a folding competent state and can subsequently be refolded by the ATP-dependent “foldase” chaperone machines (Hsp70, Hsp90, and co-chaperones) [44–47] or degraded by the ubiquin-26S proteasome after being recognized by Hsp70 interacting E3 ubiquitin ligase CHIP [48]. Holdase and foldase machines are part of a coordinated network aimed at refolding or promoting the degradation of denatured polypeptides, a phenomenon which is essential for cell survival to acute stress. Small Hsps are cytoplasmic polypeptides, except in heat shock conditions, where some of them, such as HspB1, can be recovered in the nucleus at the level of granular structures [35] that have recently been shown to contain denatured proteins [49] that are stored for subsequent degradation during heat shock recovery [50]. In the nucleus of stressed myoblast cells, HspB1 as well as HspB5 also interact with intranuclear lamin to stabilize this stress-sensitive network [51]. In addition to the modulation of mRNA translation consequently of the trapping of eIF4G initiation translation factor in insoluble heat shock granules [52], a sumoylation-mediated feedback inhibition of HSF1 transactivation is another function of these proteins in response to heat shock [53]. 

HspB1 and HspB5 are very effective to protect cytoskeletal architecture homeostasis which is deeply altered in response to thermal or oxidative stress [54, 55]. In that respect, phosphorylated small HspB1 oligomers bear an F-actin capping activity that negatively modulates F-actin fibers growth and indirectly modulates extracellular matrix organization [56–58]. Consequently to its action towards F-actin, HspB1 indirectly regulates neutrophil chemotaxis and exocitosis, neurite outgrowth [59] and maintains sustained muscle contraction [60]. Moreover, in cancer cells, HspB1 is necessary for F-actin-mediated cytokinesis and therefore, interferes with the accumulation of giant polynucleated cells [61]. HspB1 and HspB5 also stabilize microtubules [62–64] while HspB5 has been described to be efficient towards intermediate filaments, particularly in muscle cells, where it associates with desmin [65, 66]. 

Another property of HspB1 and HspB5 is their ability to protect cells through an intriguing antioxidant property that decreases the levels of intracellular reactive oxygen species and nitric oxide and concomitantly upholds glutathione in its reducing form as well as mitochondrial membrane potential (ΔΦm) [30, 67–75]. Consequently, damages such as protein oxidation, lipid peroxidation, and cytoskeleton architecture disruption are attenuated [68–70]. Moreover, the positive effect towards ΔΦm provides the cell with an increased level of ATP production that stimulates the activity of ATP-dependent foldase chaperones. 

To eliminate irreversibly damaged polypeptides, particularly the oxidized ones that cannot be refolded, HspB1 and HspB5 can trigger their degradation independently of the Hsp70-CHIP machine. Indeed, they can stimulate ubiquitination or, as in the case of HspB5, directly interact with the proteasome [76–79]. HspB8, which interacts with irreversibly altered proteins, can trigger the macroautophagy machinery, an ultimum mechanism to eliminate aggregated polypeptides generated by heat shock [80] or oxidative stress [81, 82], through a further association with Bag3 [83, 84]. 

## 3. Constitutively Expressed Small Hsps Maintain Protein Folding Homeostasis 

Studies performed in different organisms have revealed an important property of small heat shock proteins, that is, their ability to be expressed in the absence of apparent stress in specific tissues of developing and adult organisms [85–90] (see Figure 1(c)). For example HspB1, which is highly abundant in muscles, is expressed in almost all tissues. In contrast, HspB4 (alphaA-crystallin) is almost exclusively present in lens cells while HspB5 (alphaB-crystallin), which associates with HspB4 to form the lens alpha-crystallin complex, is also constitutively expressed in tissues with high rates of oxidative metabolism, such as the heart, skeletal muscle fibers, brain, and kidney. The early phase of many differentiation processes is another example in which a high level of HspB1 is transiently expressed [91–98], and where this chaperone plays an essential role [99, 100]. One hypothesis could be that HspB1 secures differentiating cells from the toxicity of proteins that are of no more use or have generated inappropriate interactions. In that regard, HspB1 could participate in the mechanism that counteracts tendency of these proteins to form junk protein structures that could aggregate before they get degraded [97, 101]. On the other hand, it is not excluded that HspB1 could hold and protect essential polypeptides during the transient hostile intracellular environment of differentiating cells. As for example, cytoskeleton whose structure can be deeply modified during cell differentiation.

## 4. Small Hsps Are Beneficial in Protein Conformational and Inflammatory Diseases

Numerous studies have reported that elevated levels of constitutively expressed HspB1 and HspB5 are observed in pathological cells in which protein folding homeostasis is impaired by the accumulation of pathological proteins that are prone to aggregate, such as *α*-synuclein, *β*-amyloid peptide as well as polyQ mutants of huntingtin polypeptide that are responsive of Parkinson's, Alzheimer's and huntington, neurodegenerative diseases, respectively. HspB1 and/or HspB5 accumulate in cortical Lewy bodies, Alzheimer disease plaques, neurofibrillary tangles, Rosenthal fibers of Alexander's-disease, Creutzfeldt-Jakob altered neurones as well as in synuclein deposit associated to Parkinson's disease or myopathy-associated inclusion body [102–106]. HspB1 and HspB5 stimulate, through their holdase activity, the cellular resistance by attenuating aggregates formation, as for example, in myocardial infarction and cerebral ischemia [107, 108] (see Table 1). Consequently, they have been described as being able to promote cardioprotection [109] and to enhance nerve survival [110]. Similarly, overexpression of HspB6, HspB7, HspB8 as well as HspB1 can independently protect against tachycardia remodeling [111, 112]. By doing so, these proteins provide a beneficit that helps cells to counteract the development of pathological process that could lead to cardiomyopathic, neurodegenerative, myopathic, cataract, and retina diseases [73, 113–118]. The *in vivo* protective activity of these proteins as potent suppressors of cell degeneration was further confirmed in transgenic mice overexpressing HspB1 that are strongly protected against myocardial infarction and cerebral ischemia [107, 108]. These facts were also confirmed by the discovery of mutations in HspB1, HspB5, and HspB8 genes that inhibit their chaperone activity and provoke human diseases such as inherited peripheral and motor neuropathies, amyotrophic lateral sclerosis (ALS), axonal Charcot-Marie-Tooth disease, myofibrillar myopathies, cardiomyopathies, and cataracts [119–125]. The name *α*B-crystallinopathies has been given to the pathologies induced by mutations in HspB5. The protection may rely, at least in part, on the ability of these chaperones to specifically induce the sequestration of toxic protein oligomers [126]. Among the other members of the small Hsp family, HspB8 has the particular property to block polyglutamine (polyQ) huntingtin inclusion formation suggesting that it maintains aggregation prone polypeptides in a soluble state competent for rapid degradation. The C-terminal domain of HspB8 appears essential for this function [33]. HspB7 is even more potent since it not only suppresses polyQ aggregation, but also prevents polyQ-induced cellular toxicity; however, unlike HspB1, it does not improve the refolding of heat-denatured polypeptides [127]. 

Through their ability to act as antioxidant molecules [67, 73, 74, 113, 128–130], HspB1 and HspB5 can be highly beneficial to cells expressing aggregated polypeptides. Indeed, oxidative stress is often a common feature of cells bearing aggregated polypeptides [131, 132] and in several of the above described diseases the production of abnormally high levels of deleterious intracellular reactive oxygen species has been detected [130, 132–138]. This is particularly the case in cells expressing pathological huntingtin, *β*-amyloid, or *α*-synuclein polypeptides which are iron/copper binding or metal homeostasis modulating polypeptides [139, 140] that can act as catalyzers and disregulate the hydroxyl radical generating Fenton reaction [141, 142]. Oxidative stress may then alter mitochondrial and proteasome function and aggravate protein aggregation [73, 113, 130, 143, 144].

Inflammatory pathologies, such as asthma, are other examples, where the antioxidant property of small Hsps has a beneficial protective role [145–149]. Indeed, through modulation of intracellular redox state and TAK-1 activity, these proteins interfere with tumor necrosis factor (TNF*α*) signaling pathways and therefore, negatively modulate inflammation processes [145, 147]. Moreover, HspB1 has been described to suppress skeletal muscle atrophy through its interaction with the activating kinases IKK-*α* and IKK-*β* of the transcription factor NF-*κ*B [148]. One can also cite ischemic-related stroke injuries and alcoholic liver diseases characterized by the presence of Mallory bodies [108, 150]. These observations suggest crucial roles of HspB1 and HspB5 in inflammatory processes.

## 5. Small Hsps Are Antiapoptotic Proteins

Apoptosis, which differs from necrotic cell death, by being a genetically programmed process that requires energy, is negatively modulated by constitutively expressed Hsps. Indeed, in contrast to cells exposed to environmental insults, such as heat shock, where Hsps are synthesized to fight against the damaging effects of stress, no upregulation of Hsps expression occurs in cells committed to apoptosis. The reason is that a cell undergoing apoptosis does not fight against its own decision to commit suicide. The problem exists because Hsps, and particularly HspB1 and HspB5, are often constitutively expressed, particularly in human cancer cells, where they counteract an apoptotic process decided by the cell. In this type of cell death which does not induce the accumulation of misfolded polypeptides, HspB1 and HspB5 interact with specific protein targets located along the signal transduction pathways activated by death receptors [151–155] as well as both upstream and downstream of mitochondria [156–162]. Complex and signal transduction-dependent structural reorganizations of HspB1 phosphorylation/oligomerization are observed in cells committed to apoptosis [39, 163] suggesting that this chaperone has multiple strategies to counteract apoptosis. Structural changes are probably needed to allow HspB1 to interact with specific targets. Among them, one can cite: cytochrome c [157, 159], procaspase-3 [61, 152], Daxx [151], Stat3 [164], eIF4E [165], F-actin [159], HDAC6 [61], Stat2 [61], PTEN [166], and the cell survival kinase Akt [153, 155, 167, 168] that indirectly antagonizes Bax-mediated mitochondrial damages [162] and PEA-15-dependent Fas-induced apoptosis [169]. In addition to sharing some of HspB1 antiapoptotic mechanisms, such as caspase-3 maturation inhibition [154, 170, 171], HspB5 has specific ways to interfere with apoptosis. For example, it blocks the translocation to the mitochondria of the anti-apoptotic polypeptides Bax and Bcl-xs [160] and inhibits the activation of the proto-oncogene RAS [161]. Both HspB5 and HspB4 also modulate Akt, PKC*α*, and Raf/MEK/ERK pathways [172]. However, it cannot be concluded that all small Hsps are anti-apoptotic proteins *per se* since in some circumstances they can have the reverse effect: for example, HspB5 phosphorylated at the level of serine59 is proapoptotic since it prevents Bcl-2 translocation to mitochondria [173]. Moreover, depending on the cell type, HspB8 has pro- or anti-apoptotic activity.

## 6. Deleterious Effect Mediated by Small Hsps Expression in Human Cancer Pathologies

Many cancer cells express high loads of Hsps, such as HspB1 and HspB5; a phenomenon which increases their resistance to numerous deleterious agents and conditions [174–177] (see Table 1). One attractive, but still not proven, mechanism to explain a phenomenon linked to increased levels of HSF1 expression [178] is the “addiction to chaperones” hypothesis [177, 179]. Addiction could be caused by profound alterations in protein homeostasis resulting from mutant proteins that accumulate in cancer cells [178, 180]. So, contrasting to their beneficial role in degenerative and inflamatory diseases, their ability to protect cancer cells could be highly deleterious on a patient point of view. In that respect, HspB1 and HspB5 are essential for the growth of cancer cells and protect them against apoptotic or other types of death triggered by the immune system in the aim of their elimination [154, 169, 181–184]. They also provide cancer cells with the ability to counteract host anticancer response, such as senescence. This leads to aggressive cell growth [185, 186], metastasis formation, dissemination [187–190], and poor prognosis [174, 175]. Many studies have tried to decipher the mechanism that allow HspB1 to trigger tumor progression and metastasis. In that regard, several observations have already been made. For example, HspB1 can indirectly modulate extracellular matrix organization [56–58] through the stimulation of metalloproteinase type 2, an enzyme that efficiently digests the matrix surrounding tumor masses [191]. In addition, it can modulate cadherin-catenin cell adhesion polypeptides consequently to its interaction with cytoplasmic *β*-catenin [192]. More recently, HspB1 has been proposed to participate in the maintenance of breast cancer stem cells through regulation of the epithelial to mesenchymal transition process [193]. 

Another worth noting negative point concerns HspB1 ability to provide cancer cells with resistance to many anti-cancer drugs, which in turn, unfortunately, stimulates HspB1 expression [194–198]. Hence, high levels of HspB1 expression correlate with a poor clinical outcome of gastric, uterine, breast, prostate, ovarian, and head/neck cancers as well as of tumors from the urinary and nervous systems. 

Other members of the family, such as HspB4 and HspB5, are also deeply involved in cancer biology. The first intriguing observation concerns HspB5 expression which transforms immortalized human mammary epithelial cells that can, subsequently to their injection in nude mice, develop in invasive mammary carcinomas that have the same aspect as basal-like breast tumors. At the molecular level, it has been found that HspB5-mediated growth of human breast basal-like tumor cells is epidermal growth factor (EGF)- and anchorage-independent. It increases cell migration and invasion through a constitutive activation of the MAPK kinase/ERK (MEK/ERK) pathway [199]. Hence, in addition to its anti-apoptotic property, HspB5 has the surprising ability to behave as an oncoprotein and consequently breast tumors expressing high levels of this protein are linked to short patient survival [200]. Contrasting with these observations, HspB4 expression in pancreatic cancer is a negative regulator of tumor development that has a good prognosis value [201].

## 7. Dual Role of Extracellular sHsps 

The function of heat shock proteins goes beyond their intracellular localization and chaperone role since an increasing number of studies have recently described that, under normal physiological and stress conditions, a fraction of the cellular content of several Hsps, including the small Hsps, is recovered into the extracellular space, where they activate signaling pathways [202, 203]. The phenomenon, which is not related to cell injury or necrotic events, suggests a novel role of Hsps as universal proinflammatory intercellular “danger” signalling molecules. Hence, the classical role(s) of these highly conserved and ubiquitously expressed families of polypeptides is actually critically reevaluated. In that regard, Hsp60 was the first heat shock protein reported to be outside cultured cells [204]. Then, several studies demonstrated that Hsp70 and Hsp60 were localized on the cell surface [205–207], released in the extracellular milieu [208–212], and detected in the serum of normal and stressed individuals, together with circulating antibodies against these proteins [205, 213–215]. The level of Hsps in the serum of human individuals is highly variable and depends on multiple factors such as exercise [216], psychological stress [217], and diseases [211, 218]. This discovery has opened new roads of investigation aimed at understanding the role played by extracellular Hsps. It was first concluded that extracellular Hsps have a wide variety of functions towards neighboring cells including the possibility of being a danger signal to the immune system [219]. For example, it has been shown that in the brain, Hsp70 is released from glial cells and can subsequently interact with neurons and stimulate their ability to cope with stressful conditions [212]. Extracellular Hsp70 has also been reported to reduce neuronal polyglutamine toxicity and aggregation [220] and to change behavior in rats [221]. Circulating Hsp70 levels also predict, and may attenuate, the development of atherosclerosis in subjects with established hypertension [222]. On the opposite, in patients with colorectal cancer without distant metastasis, serum level of Hsp70 is associated with high mortality [223]. Another aspect of Hsp70 and Hsp60 deals with their immunogenicity and ability to activate dendritic cells as well as the production and secretion of cytokines [222, 224, 225]. Moreover, stimulation of both innate and adaptive forms of antitumor immune responses can be achieved through tumor-derived extracellular Hsp70-, Hsp90-, and gp96-peptide complexes that bind receptors on antigen presenting cells (APCs) and deliver tumor-specific antigens to major histocompatibility complex (MHC) class I molecules on the surface of such cells [207, 226–228]. Such antigen cross-presentation interactions form the basis for the “Hsp-based anticancer vaccines technology” [174, 229, 230] whose potency depends on the ability of Hsps to chaperone tumor antigenic peptides that stimulate antitumor immune responses through Hsp receptors [226, 231, 232].

Small Hsps have often been described as membrane associated proteins [233–235], and several recent reports point to their presence in the extracellular milieu. However, it is not yet known whether they could, similarly to the high molecular weight Hsps, elicit an immune response aimed at killing cancer cells through their association with immunogenic peptides. Despite this point, several positive and negative (for a patient point of view) functions of these extracellular proteins have already been reported (see Table 1). One interesting example concerns the atheroprotective effect of circulating HspB1 [236]. This protein, which has been known for quite a while to be an estrogen receptor beta (ERbeta)-associated protein, was noted for its role as a biomarker for atherosclerosis. The key experiment was the crossing of transgenic mice overexpressing HspB1 with apoE^−/−^ mice that develop atherosclerosis when fed a high-fat diet. This experiment revealed a reduction in atherosclerotic lesion area in apoE^−/−^-HspB1 mice compared to apoE^−/−^ mice. An interesting point of the phenomenon was its estrogen receptor-beta dependence. Indeed, it occurred only in females, where it correlated with a 10-fold higher level of circulating HspB1 compared to males. Moreover, there was a remarkable inverse correlation between circulating HspB1 levels and intensity of the lesions area. The atheroprotective activity of HspB1 was further confirmed by the inhibition of macrophage acLDL uptake and competition for the scavenger receptor by exogenous HspB1 added to culture media as well as by the decreased release of the proinflammatory cytokine interleukin-1*β* (IL-1*β*) and the increased release of the anti-inflammatory cytokine interleukin-10 (IL-10). Hence, the ovarian hormones mediated atheroprotective activity of HspB1 appears to be a consequence of its ability to compete for the uptake of atherogenic lipids and cholesterol and to attenuate vascular inflammation [237]. Based on the strong experimental evidence that ovarian hormones have a favorable effect on vessel wall homeostasis, HspB1 can therefore, be considered as an interesting target that leads to the development of therapeutic drugs that can be used in replacement of the unfavorable risk-benefit profile of estrogen in vascular diseases preventing therapy of postmenopausal women [238]. It is also well-known that, in men and women, HspB1 shows an attenuated expression in human coronary arteries as the extent of atherosclerosis progresses. Up-regulation of HspB1 blocks this progression as demonstrated in transgenic mice overexpressing this protein. In a mechanistical point of view, it has recently been reported that recombinant HspB1 added to macrophages activates NF-*κ*B and consequently changes the balance in the expression of key pro- and anti-inflammatory cytokines and antagonists of inflammation. These HspB1 triggered NF-*κ*B-dependent signalings may explain the favorable net effect of HspB1 on the vessel wall [239]. Another example deals with the cardiovasculature which is probably the most exposed body system to stress. Hsps in the heart are known to be cardioprotective and their secreted counterparts play essential roles in the function of the cardiovascular tissues. In that respect, a positive action of circulating HspB1 has been demonstrated which deals with its anti-inflammatory capability that attenuates cardiovascular pathology [240]. On the negative side, high levels of HspB1 cell surface expression correlates with tumor growth and ability to metastasize [241]. Moreover, high levels of circulating HspB1 are also associated with tumor progression and increased postinjury infection [242–244]. By altering monocyte-derived dentritic cells to mediate immunosuppression, extracellular HspB1 has been proposed to have immunoregulatory activities that could contribute to immunopathology. Several other examples exist concerning disease-induced changes in the level of circulating HspB1; however, it is still unknown whether the phenomenon can be beneficial or not for the patient. For example, increased levels of circulating HspB1 are associated with micro- and macrovascular complications in type 1 diabetic patients and considered as a novel marker for diabetic neuropathy [245]. Hence, circulating extracellular small Hsps can have pathology-dependent dual roles similarly to their intracellular counterparts. The role of these extracellular proteins in normal physiological conditions is still not known, and speculations are open.

Because Hsps are intracellular proteins, a mechanism for their release into extracellular space must exist but remains obscur. First, it should be noted that Hsps are devoid of secretion signals, and their release is not blocked by inhibitors of ER-Golgi pathway, such as brefeldin A. Two mechanisms can be considered as follows: passive release consequently to necrotic cell death, trauma, or infection with lytic viruses and nonclassical active release. In that respect, active release can be triggered by agents, such as proinflammatory cytokines [208]. Recent observations suggest that, at least in the case of Hsp70, insertion of this Hsp into the plasma membrane requires inverse evagination, and its release from the cell is in a membrane-associated form (i.e., exosome) [212, 246, 247]. More precisely, the mechanism may involve surface membrane lipid rafts and the shedding of exosomes vesicles containing cytoplasmic constituents [203, 212, 248]. Strikingly, the tumor exosome-associated form of Hsp70 appears drastically more active than the free recombinant Hsp70 to stimulate macrophages [247] and natural killer cells [249]. Concerning the small Hsps, an interesting observation has been made in breast cancer patients with lymph node metastases. These patients show increased levels of circulating HspB1-positive microparticles [250] as well as microparticles containing annexin V, Her2/neu, and BCRP1 (Breast Cancer Resistance Protein 1). The origin of these microparticles is unknown, but they could be exosomes, hence, suggesting that HspB1 is released from cancer cells by a mechanism close to that of Hsp70.

Concerning the target receptors that are recognized by Hsps, many cell surface proteins have been described as possible candidates; however, they are characterized by low-affinity interactions with Hsps. Nevertheless, two groups have been defined that are weakly or indirectly recognized by Hsp70, Hsp60, and a member of the Hsp90 family, gp96: the Toll-like receptors (TLRs) and scavenger receptors (SRs) [251]. The TLRs are major pattern recognition receptors (PRRs). TLR2 and TLR4 are Hsp60, Hsp70, and gp96 receptors that activate NF-*κ*B [252, 253]. In addition, CD14, a human monocyte cell surface polypeptide which couples LPS exposure to TLR4 activation, is also required for Hsp70-mediated induction of TNF*α*, IL-1*β* and IL-6 [254]. CD14 is also recognized by Rhizobium leguminosarum chaperonin Hsp60.3 to trigger cytokine production [255]. These observations further demonstrate that Hsps can have a dual role as chaperone and cytokine. SRs are receptors for chemically modified forms of lipoproteins, and some of them can interact at high affinity with Hsp70, Hsp60, gp96, and Hsp90 [256–258]. The effects mediated by these interactions are complex and can have opposite effects. For example, LOX-1 mediates Hsp70 immunogenicity and antigen presentation [256], while gp96 binding to SR-A1 is immunosuppressive [259]. Of interest, a recent report has linked the inhibition of immune antitumoral activity to exosomes bearing Hsp70 when they interact with Toll like-receptor-2 of myeloid-derived suppressive cells (MDSCs); a phenomenon which inhibits the development of antitumoral response [260]. Taken together, these observations point to the complexity of the role played by extracellular Hsps towards their already described receptors. Unfortunately, no cell surface polypeptides have yet been characterized as putative small Hsps cell surface receptors. 

## 8. Circulating sHsps Autoantibodies 

As mentioned above, fascinating observations have been made concerning circulating autoantibodies against Hsps which are detected under normal conditions but seem to be more abundant in response to environmental or occupational stress and in a number of diseases [261]. As immunodominant molecules, Hsps can stimulate the immune system, leading to the production of autoantibodies recognizing epitopes shared by microbial and human Hsps. Surprisingly, such antibodies can regulate the inflammatory response positively or negatively. One example concerns breast cancer cells which express elevated levels of Hsps, a phenomenon that quite often correlates with reduced survival. So, does this provoke a generalized immune response towards Hsps? The answer is no, since serum HspB1 and Hsp90 autoantibodies show elevated levels but not Hsp70 autoantibody. Moreover, contrasting with the reduced survival associated to Hsp90 antibody, antibody to HspB1 has the surprizing property to correlate with an improved rather than a reduced survival. This leads to the conclusion that high levels of Hsps in breast cancer cells do not provoke a generalized immune response, and that Hsps serum autoantibodies have distinct associations with survival [262]. Hence, levels of circulating Hsps and anti-Hsps antibodies are now considered as useful parameters in tumor diagnosis [174]. Another example, dealing with small Hsps, concerns the presence of antibodies to HspB1, HspB5, Hsp70, and vimentin in aqueous humor of patients suffering from retinal pathologies, such as normal tension glaucoma [263, 264]. Of particular interest was the observation that exogenously applied HspB1 antibody enters human retina neuronal cells through an endocytic mechanism. This inactivates intracellular HspB1 and subsequently facilitates neuronal apoptosis [265]. Hence, it is believed that autoantibodies to small Hsps may impair cell survival in selective diseases, particularly those related to the human eye [264, 265]. In addition, it has been proposed that HspB1 is a target of the exaggerated T cell response in psoriasis and an antigenic link between psoriasis and inflammatory bowel disease, uveitis, or arteriosclerosis, which are clinically associated pathologies [266]. However, care should be taken before concluding that one fundamental property of small Hsps is to act as autoantigens. In that respect, an interesting example concerns HspB5 in multiple sclerosis [267]. In this pathology, HspB5 has been considered for many years as an autoantigen based on its effects on humoral and cellular responses. However, this statement is probably not correct since recent experiments have shown that HspB5, through its chaperone activity, can bind immunoglobulins with high affinity. This obviously refutes most of the serological data used to assign HspB5 as an autoantigen in multiple sclerosis [268]. 

Hence, extracellular Hsps and autoantibodies to Hsps are likely to act as indicators of the physiological conditions of cells. These factors can prime other cells, particularly those of the immune system, to avoid the propagation of the insult. The cellular communication mechanism for sensing extracellular Hsps has been called “the stress observation system” [203]. Depending on the pathology, this mechanism could obviously be beneficial or not to the patient. 

## 9. Small Hsps Multiple Functions Result of Their Interactions with Client Polypeptides

Small Hsps are surprizing proteins that have an incredible number of unrelated cellular functions as illustrated by the effects associated to their over- or underexpression. This may result from small Hsps interactions with a large number of client proteins that are essential to many cellular processes. In that respect, the most studied protein is HspB1; a protein known to interact with up to 34 polypeptides [176, 177]. The phenomenon is reminiscent of the already described “Hsp90/client protein concept” [269, 270]. Hsp90 is known to interact with over 200 client polypeptides (for an updated list see: http://www.picard.ch/downl-loads) in order to modulate their activity and/or half life. Hence, similar to Hsp90, HspB1, and probably other small Hsps are global regulators of cell systems [271, 272]. Some of the major clients which need to interact with HspB1 to avoid proteolytic degradation are Her2 oncogene, procaspase 3, HDM2, the histone deacetylase HDAC6, the transcription factor Stat2, and PTEN [61, 166, 183, 197]. Amongst the many clients whose activity is modified by HspB1, one can cite the translation initiation factor 4E (eIF4E) which modulates the translational initiation process, a crucial parameter for cancer cell growth and proliferation [165]. HspB1 client proteins, essential in tumorigenic and metastatic process, are nowadays actively searched for. 

How HspB1 recognizes client protein targets? Based on what is known for Hsp90, whose interactions with cochaperones and clients occur through a variety of conformational states [273, 274], HspB1 may take advantage of its complex and dynamic oligomerization-phosphorylation properties to generate structural organizations that can interact with specific protein substrates [39, 61, 176, 177, 275]. In other words, it is now believed that HspB1 is an environmental sensor, which through specific changes in its apparent native size/phosphorylation can reprogram its pattern of interacting client protein targets. Consequently, HspB1 dynamic interactome may allow cells to quickly respond and mount the more appropriate response to a particular condition or insult [39, 176]. How changes in cell physiology could modulate the structural organization of HspB1 is still an unsolved question. The phenomenon may rely, at least in part, on the complex patterns of MAPKAPK2,3-dependent phosphorylation of three serines sites located in the N-terminal domain of HspB1 [39, 176, 276, 277]. Unfortunately, no precise information is yet available concerning the structural organizations of HspB1 that recognize crucial client polypeptides. An increased complexity may arise in cells expressing several small Hsps. Indeed, these proteins can interact with each other to form multiple combinatorial oligomeric structures [278–281] that could bear new protein targets recognition abilities.

## 10. Therapeutic Approaches

It is now well established that small heat shock proteins increase cellular resistance to damages induced by stress or pathological conditions. Hence, it would be interesting to stimulate their expression to protect cells that are suffering and dying because of pathological conditions, such as those encountered in protein conformational and inflammatory diseases. The aim of this approach, by using drugs that up-regulate small Hsps holdase activity in a definite tissue, is to strengthen the cellular homeostasis protein folding and redox status machineries. These are potent systems that exist in every cells but which are limited and can be overwhelmed by pathological polypeptides [282] or drastic oxidative conditions [74, 128]. Moreover, compounds able to boost the expression of single or multiple members of the HspB family have a cardioprotective role involved in the maintenance or restoration of tissue integrity and contractile function, probably through the important role played by these Hsps towards cardiac muscle cells [111, 112]. On the flip side, such an approach could be highly detrimental in case of pathologies, where Hsps are involved in the resistance of invading pathological cells that can kill the patient, such as cancer cells. Moreover, we do not know what could be the effects of such strategies towards circulating small Hsps. In spite of these limitations, efforts are nevertheless made to discover drugs that can specifically stimulate small Hsps expression in a define tissue. One interesting example concerns the beneficial protective effect of orally administered geranylgeranylacetone in transgenic mice suffering from HspB5 mutation-dependent cardiomyopathy [283]. The effect in the heart correlated with reduced amyloid aggregates and increased HspB1 and HspB8 expression. However, what could be the effect of geranylgeranylacetone in other pathologies and particularly in primary tumors? This point should be investigated. Mimicking the holdase activity of small Hsps by drugs or peptides is an other way to tackle the problem. For example, carnosine and its acetyl derivative are effective as anticataract drugs due to their chemical chaperone ability that mimics HspB4-HspB5 holdase activity [284, 285]. Peptide aptamers that interact with small Hsps and positively modulate their activity are also interesting towards degenerating diseases since they may lead to the generation of stimulating peptidomimetic drugs [286].

In cancer pathologies, the problem associated to small Hsps expression is far more complex than in protein conformational and inflammatory diseases. At first glance, the therapeutic strategies described above which consist in stimulating small Hsps expression and/or activity are not appropriate since they would result in an increased resistance and aggressivity of cancer cells. Moreover, what is the role of circulating small Hsps and of anti-small Hsps antibodies in cancer patients? Is it beneficial or deleterious? Do small Hsps, like several other Hsps, interact with cancer-specific antigenic peptides that stimulate both innate and adaptive forms of antitumor immune responses? If this is indeed the case, care will then have to be taken to choose strategies that do not disturb this particular activity when the protective one associated to intracellular small Hsps is inactivated. 

Antisense DNA vectors [287] and more recently RNA interference (RNAi) technologies have been used to decrease the intracellular level of small Hsps and destabilize their interactome. In that respect, the most studied protein has been HspB1 whose decreased level sensitized cancer cells to apoptotic inducers, anticancer drugs, and radiations and reduced their tumorigenic potential [61, 188, 189, 196, 288, 289]. In tumor, this may lead in the degradation of HspB1 tumorigenic and metastatic client proteins. However, in other tissues, RNAi may also induce the depletion of useful proteins chaperoned by HspB1 and/or abolish HspB1 antiaggregation and antioxidative effects; phenomena that could generate pathological side effects or stimulate diseases. 

The search for less broad and more specific ways to abolish or stimulate small Hsps activity is a very difficult task since it will have to modulate, in a definite cell type, the complex formed by the targeted small Hsp with specific pathological clients or aggregated proteins. Moreover, these future procedures should not interfere with the activity of the targeted small Hsp when it is expressed in other tissues or when it interact with other clients. In the meantime, a better knowledge of the holdase activity and structure of the different small Hsps present in human cells will be required to open the road to the search of drugs that could inhibit their interactions with specific clients. This is illustrated by a recent analysis of the architecture and dynamics of complexes formed *in vitro* between an oligomeric small Hsp and client which revealed that over 300 different stoichiometries of interaction are possible [290, 291]. The specificity of the interaction of small Hsps with clients has been confirmed by two recent studies. The first one dealt with two peptide aptamers that specifically recognize different molecular surfaces of HspB1 and attenuate its antiapoptotic, antitumorigenic and cytoprotective activities [275]. The second study concerns RP101 (Bromovinyldeoxyuridine, BVDU, Brivudine), an antiviral drug that improves the efficiency of human pancreatic cancer chemotherapy through interaction with two phenylalanine residues (Phe29 and Phe33) in the N-terminal domain of HspB1. RP101 inhibits HspB1 interaction with specific procancerous binding partners and stimulates caspases activation [292].

## 11. Conclusion 

In the recent years reports dealing with the expression and involvement of small Hsps in human pathologies as diverse as neurodegeneration, myopathies, cardiomyopathies, cataracts, inflammatory diseases, and cancers have grown exponentially. Until recently, it was believed that these Hsps were specialized molecular chaperones mainly synthesized in stress conditions and whose activity was to attenuate the damages to cellular proteins by inducing their storage until they could be refolded. The recent findings clearly show that, together with other Hsps, these proteins can be constitutively expressed and have an incredible number of crucial roles in normal and pathological cells. These activities are probably linked to their abilities to recognize, interact, and modulate the activity and/or half-life of many specific protein client targets. This particular protective role of small Hsps towards protein folding can have dual consequences: (i) by helping cells to better cope with their pathological status; they can be beneficial in diseases characterized by pathological cell degeneration, (ii) by helping cells that evade death and proliferate, such as cancer cells, the activity of small Hsps can be highly deleterious. A third consequence could be towards small Hsps that are actively released by cells. It is now well established that small Hsps are therapeutic targets whose activity needs either to be stimulated or abolished depending on the pathology. To be efficient and propose strategies aimed at designing active molecules that could modulate the activity of these Hsps, future works will have to unravel the precise role of their multiple combinatorial phospho-oligomeric structures to understand their complex interactions with many specific client proteins. These studies together with structural work [293, 294] and analysis of the organization of these proteins in living cells [39] will probably allow the discovery of new drugs testable for their effectiveness in different pathologies. As described here, the use of broad drug screening or genetic techniques to invalidate the activity or expression of these proteins could appear efficient, but on the long term they may prove to be disappointing due to unsuspected side-effects. Indeed, we should keep in mind the unfortunate modest effects and lack of FDA recognition reported to date for the broad inhibitors of Hsp90 chaperone activity in most cancer clinical trials [295].

## Figures and Tables

**Table 1:** Beneficial and deleterious roles of small Hsps. Schematic illustration of the dual role of intracellular and extracellular small Hsps as well as autoantibodies against these proteins.

Beneficial role		Deleterious role
Neurodegenerations		
Myopathies		
Cardioprotection	Intracellular small Hsps	
Cataracts		Cancers
Strokes		
Asthma		
Inflammatory disease		

Atherosclerosis Antiinflammatory effects Anticardiovascular effects	Extracellular, circulating small Hsps	Tumor progression Metastasis Increased postinjuries infection Immunosuppression

Avoid propagation of insults		Facilitate retinal apoptosis
	Autoantibodies	Psoriasis
		Autoimmune diseases
